# Royal Medical and Chirurgical Society

**Published:** 1897-05-01

**Authors:** 


### ROYAL MEDICAL AND CHIRURGICAL SOCIETY.

A considerable amount of interest always attaches to the isolation of a group of symptoms and its association with more or less definite pathological changes in such manner as to establish the existence of what may be termed a new disease.

The last meeting of the Royal Medical and Chirurgical Society was occupied in the discussion of a paper read at the previous meeting by Dr. Walter Carr, in which he described the symptoms and the pathological appearances met with in certain cases of non-tuberculous posterior basic meningitis in infants. Such a large proportion of all cases of meningitis in children are tuberculous that we are apt almost to ignore the existence of other forms. The temptation is strong to look upon them as merely aberrant forms of the more common disease, for whereas the tuberculous disease is one running a tolerably definite course, the other and rarer varieties present the greatest diversity both as to symptoms, course, and morbid appearances, and after all, speaking generally, seem more nearly linked by symptoms to the tuberculous type than they are by morbid appearances to each other. Amid this chaos, however, Dr. Carr singled out a certain number of cases which presented a fairly definite course and symptomatology, and tolerably uniform pathological changes, and which were therefore probably due to some one definite cause, although this had as yet eluded observation. It occurred almost exclusively in infants, mainly affected the posterior part of the base of the brain, was entirely independent of tuberculous disease, ran a subacute or chronic course with fairly characteristic symptoms, and presented very definite and constant *post-mortem* appearances. All the cases occurred in previously healthy infants under a year old; the onset was gradual in some, sudden in others; but, in all, the most constant and characteristic symptoms were severe vomiting, extreme retraction of the head, and stupor passing into coma of remarkably long duration—generally several weeks—the excessive retraction of the head persisting to the last. The cases all terminated fatally, none in less than five weeks, whilst some lived for three months or even longer. At the necropsies, inflammation of the pia arachnoid was found over a very definite area at the base of the brain, hydrocephalus was found in all the cases, and in some closure of the openings between the fourth ventricle and the sub-arachnoid space. In no instance was any sign of tuberculous disease discovered. Under the head of diagnosis Dr. Carr showed that this disease had to be distinguished from other forms of meningitis, especially the tuberculous form. The possibility of some relationship with epidemic spinal meningitis was also considered.

The discussion was joined in by Dr. Barlow, Dr. Lees, Mr. Ballance, Dr. Abercrombie, Mr. Raymond Johnson, and others.

The anatomical relations of the parts were considered, and the possible connection of the condition described with various other influences, which might be looked upon as causes, even if only predisposing ones, was discussed.

In regard to treatment, considerable stress was laid on the possibility of relieving the effusion by operative means, as had been done in some of the cases which were related. It was pointed out, however, that to be of real service such operative interference must be undertaken early, the object being not merely to drain a hydrocephalic effusion, but by the relief of tension to prevent the spread of a specific infection.

